# Tristetraprolin targets *Nos2* expression in the colonic epithelium

**DOI:** 10.1038/s41598-019-50957-9

**Published:** 2019-10-08

**Authors:** Melanie A. Eshelman, Stephen M. Matthews, Emily M. Schleicher, Rebecca M. Fleeman, Yuka Imamura Kawasawa, Deborah J. Stumpo, Perry J. Blackshear, Walter A. Koltun, Faoud T. Ishmael, Gregory S. Yochum

**Affiliations:** 1 Department of Biochemistry & Molecular Biology, Pennsylvania State University College of Medicine, Hershey, PA 17033 USA; 2 Department of Surgery, Division of Colon & Rectal Surgery, Pennsylvania State University College of Medicine, Hershey, PA 17033 USA; 3 The Institute for Personalized Medicine, Pennsylvania State University College of Medicine, Hershey, PA 17033 USA; 4 Department of Pharmacology, Pennsylvania State University College of Medicine, Hershey, PA 17033 USA; 5 Signal Transduction Laboratory, National Institute of Environmental Health Sciences, Research Triangle Park, NC 27709 USA; 6 Departments of Medicine & Biochemistry, Duke University Medical Center, Durham, NC 27710 USA; 7 Department of Allergy & Sleep Medicine, Mount Nittany Medical Group, State College, PA 16803 USA

**Keywords:** RNA, Cell growth

## Abstract

Tristetraprolin (TTP), encoded by the *Zfp36* gene, is a zinc-finger protein that regulates RNA stability primarily through association with 3′ untranslated regions (3′ UTRs) of target mRNAs. While TTP is expressed abundantly in the intestines, its function in intestinal epithelial cells (IECs) is unknown. Here we used a cre-lox system to remove *Zfp36* in the mouse epithelium to uncover a role for TTP in IECs and to identify target genes in these cells. While TTP was largely dispensable for establishment and maintenance of the colonic epithelium, we found an expansion of the proliferative zone and an increase in goblet cell numbers in the colon crypts of *Zfp36*^ΔIEC^ mice. Furthermore, through RNA-sequencing of transcripts isolated from the colons of *Zfp36*^fl/fl^ and *Zfp36*^ΔIEC^ mice, we found that expression of inducible nitric oxide synthase (*iNos* or *Nos2*) was elevated in TTP-knockout IECs. We demonstrate that TTP interacts with AU-rich elements in the *Nos2* 3′ UTR and suppresses *Nos2* expression. In comparison to control *Zfp36*^fl/fl^ mice, *Zfp36*^ΔIEC^ mice were less susceptible to dextran sodium sulfate (DSS)-induced acute colitis. Together, these results demonstrate that TTP in IECs targets *Nos2* expression and aggravates acute colitis.

## Introduction

A single layer of epithelial cells lines the intestines and forms a physical barrier to protect underlying tissue from the microbiota and noxious contents of the lumen^1^. In the small intestine, the epithelium is arranged into villi, that protrude into the lumen and increase epithelial surface area, and invaginations, known as crypts of Lieberkühn, that form a protective niche for intestinal stem cells. The colon is void of villi but maintains the protective crypt structures. Within the colonic crypts, intestinal stem cells divide in order to self-renew and also produce transit-amplifying progenitor cells^2^. As transit-amplifying cells divide, they migrate up the crypt axis toward the lumen of the colon and differentiate along either an absorptive or secretory lineage. The absorptive lineage consists entirely of enterocytes, which absorb nutrients, vitamins, and water from the lumen. The secretory lineage consists of mucus-secreting goblet cells, hormone-secreting enteroendocrine cells, and tuft cells^3^. The entire epithelium is replaced every three to five days, making the intestines one of the most regenerative organs in the body. Thus, IEC proliferation and differentiation must be precisely coordinated to maintain homeostasis.

There is mounting evidence demonstrating an integral role for post-transcriptional gene regulation in intestinal homeostasis and disease, including colitis and colorectal cancer^4^. RNA-binding proteins can either degrade or stabilize transcripts by associating with 3′ UTRs of target transcripts and recruiting multiprotein complexes. One such protein is tristetraprolin (TTP; also known as TIS11, NUP475, and G0S24), which is encoded by the *Zfp36* gene^5^. TTP is the prototypical member of a small family of tandem CCCH zinc finger RNA-binding proteins that also includes ZFP36L1 and ZFP36L2, also known as TIS11B and TIS11D, respectively^5–7^. TTP binds AU-rich elements in the 3′ UTR of target transcripts through the consensus binding motif, AUUUA^5^. Once bound, TTP stimulates mRNA degradation through recruitment of the DCP2/XRN1/EDC3 decapping complex and/or the NOT1/CAF1/CCR4 deadenylation complex^8^. TTP has primarily been studied in macrophages, and many established TTP target genes encode immune modulators, such as TNFα^5^. Consequently, germline TTP knockout mice develop a severe inflammatory syndrome and fail to thrive^9^. Despite robust TTP expression in the intestine, little is known about its function in IECs and whether it contributes to intestinal homeostasis or digestive diseases^10^.

Nitric oxide synthases (NOS) catalyze the conversion of arginine to citrulline and nitric oxide (NO)^11^. Unlike family members, neuronal NOS (nNOS or NOS1) and endothelial NOS (eNOS or NOS3), which are constitutively expressed in neurons and endothelial cells, respectively, expression of the third member, inducible NOS (iNOS or NOS2), is not tissue restricted and is induced by various cytokines or bacterial cell wall components^11,12^. Indeed, bacterial infection has been shown to increase expression of NOS2 in human colonic epithelial cells^13^. NOS2 localizes to the apical pole of IECs and is thought promote NO release into the lumen, where it has cytotoxic effects on bacteria^14,15^. While NOS2 and NO have many beneficial functions in the intestines, NOS2 levels must be precisely controlled to prevent pathogenic levels of NO^16–18^.

In this study we sought to identify TTP targets in IECs and to uncover the role for epithelial TTP in intestinal homeostasis and acute colitis. We found that TTP is largely dispensable for the maintenance of the colonic epithelium, yet its loss alters the expression of a variety of transcripts. Indeed, we identified increased *Nos2* expression in TTP-depleted IECs. Furthermore, we demonstrated that *Nos2* is targeted by TTP through interactions with its 3′ UTR. These post-transcriptional alterations in TTP-depleted IECs protect mice from a model of acute colitis.

## Results

### Tissue-specific removal of TTP in the colonic epithelium

**Figure 1:**
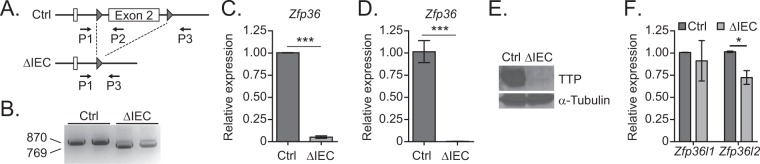
Tissue-specific removal of TTP in the colonic epithelium. (**A**) Schematic of Cre-mediated deletion of exon two of *Zfp36* from the IECs of ΔIEC mice. Primers used for genotyping are indicated. (**B**) PCR analysis of genomic DNA isolated from full thickness colonic tissue to assess the efficiency of Cre-mediated *Zfp36* deletion in Ctrl and ΔIEC mice. The floxed and deleted alleles produce 870 bp and 769 bp PCR products, respectively. (**C**,**D**) RT-qPCR analysis of transcripts isolated from full thickness colonic tissue (**C**) and purified colonic epithelium (**D**) from Ctrl and ΔIEC mice. Expression levels are normalized to *β-actin*. (**E**) Western blot analysis of TTP protein levels in full thickness colon tissue from Ctrl and ΔIEC mice. (**F**) RT-qPCR analysis of *Zfp36l1* and *Zfp36l2* transcripts in purified colonic epithelium from Ctrl and ΔIEC mice. Expression levels are normalized to *β-actin*. Error bars represent SEM (*P < 0.05, ***P < 0.001). The original uncropped images of B and E are shown in Fig. S4.

We employed a genetic approach to remove *Zfp36*, which encodes TTP, in the intestinal epithelium. Specifically, we bred *Zfp36*^*fl/fl*^ mice, that contain LoxP sequences flanking exon two of *Zfp36*, with *Villin*:*Cre* mice where expression of the CRE recombinase is restricted to the intestinal epithelium through sequences derived from the *Villin* promoter that become active at E12.5 (Fig. 1A)^19,20^. We refer to these mice as *Zfp36* deleted in intestinal epithelial cells (*Zfp36*^ΔIEC^), or ΔIEC for simplicity. Expression of TTP in the IECs was not essential as ΔIEC mice were born at expected Mendelian ratios and were otherwise indistinguishable from non-ΔIEC littermates (not shown).

Before characterizing the intestines, we assessed the efficiency of *Villin:Cre* mediated deletion. We purified genomic DNA from colonic sections, conducted PCR with primers flanking the *Zfp36* targeted site, and found that the majority of *Zfp36* alleles from ΔIEC colon tissue contained deletions of exon two (Fig. 1B). Consequently, *Zfp36* expression was dramatically reduced in RNAs isolated from full-thickness tissue and was reduced more than 99% in purified colonic epithelium from ΔIEC mice (Fig. 1C,D). This reduction was apparent at the protein level as very little TTP was detected by western blot analysis of ΔIEC colonic lysates (Fig. 1E). These results indicate the efficacy of our genetic approach to remove TTP expression from the colonic epithelium. Furthermore, expression of *Zfp36* paralogs, *Zfp36l1* and *Zfp36l2* was largely equivalent in knockout and control mice, suggesting that TTP depletion does not lead to an aberrant increase in the expression of other TTP family members (Fig. 1F).

### TTP loss increases IEC proliferation and the number of goblet cells in the colonic epithelium

**Figure 2:**
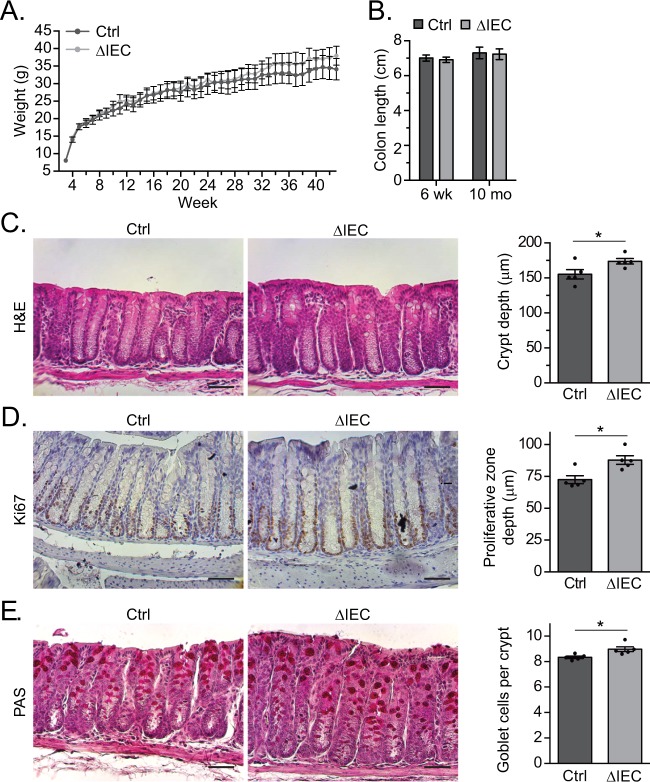
TTP loss increases IEC proliferation and the number of goblet cells in the colonic epithelium. (**A**) Body weights of Ctrl (n = 7; 5 female and 2 male) and ΔIEC (n = 7; 5 female and 2 male) mice aged 3 weeks to 10 months old. (**B**) Colon lengths from Ctrl (n = 7) and ΔIEC (n = 7) mice aged 6 weeks or 10 months. (**C**) H&E, (**D**) Ki67, and (**E**) PAS staining of colon sections from 6-week-old Ctrl and ΔIEC mice. Scale bars indicate 50 μm. To the right of each panel, crypt depth, proliferative zone depth, and the number of goblet cells per crypt were quantified from 25 crypts per mouse in n = 5 mice per genotype. Each point indicates the average for an individual mouse. Error bars represent SEM (*P < 0.05).

To begin to assess whether deletion of TTP in the IECs affected the integrity of the colonic epithelium, we first monitored weights of male and female control (*Zfp36*^*fl/fl*^) and ΔIEC mice over a period of 42 weeks. We detected no significant differences in weight gain indicating that knockout mice did not manifest gross deficiencies in intestinal function (Fig. 2A). Furthermore, the lengths of excised colons from control and ΔIEC mice were similar in both juvenile (6 wk) and adult mice (10 mo) (Fig. 2B).

We next determined whether TTP removal affected the architecture of the intestinal epithelium. We first evaluated the small intestines and found that the epithelial architecture and cellularity of the small intestines from ΔIEC mice were largely unperturbed (Fig. S1A–F). Next, we evaluated the colons and found that those from ΔIEC mice displayed deeper crypts in comparison to control littermates (Fig. 2C). Transit amplifying cells produced by stem cells at the base crypt proliferate and migrate up the crypt axis towards the lumen^2^. By staining sections with anti-Ki67 antibodies, which mark proliferating cells, we detected an expansion of the proliferative zone in ΔIEC colons (Fig. 2D). Previously, we and others have shown that an increase in the numbers of proliferating cells can alter the composition and numbers of cells comprising the differentiated compartment^21^. Using periodic-acid Schiff (PAS) staining to detect goblet cells, we noted a slight increase in the number of these cells per crypt in the colons of knockout mice (Fig. 2E). Thus, while TTP deletion increased numbers of proliferative and goblet cells in the colon, overall colonic architecture was largely intact.

### TTP suppresses expression of inducible nitric oxide synthase in the colonic epithelium

**Figure 3:**
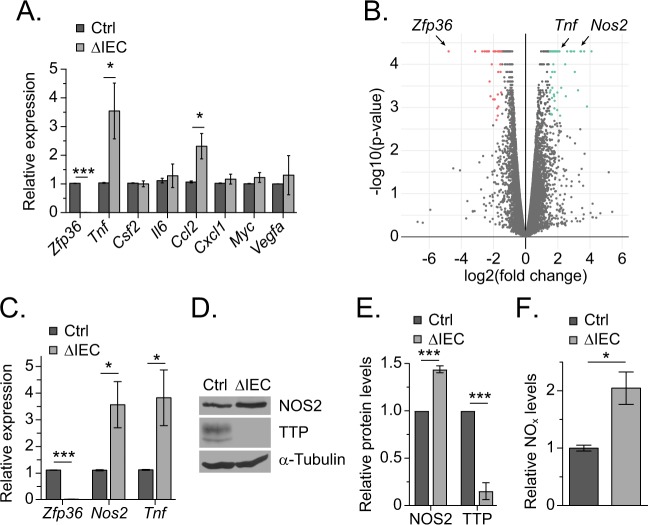
TTP suppresses expression of inducible nitric oxide synthase in the colonic epithelium. (**A**) RT-qPCR analysis of transcripts corresponding to established TTP target genes in purified colonic epithelium from Ctrl (n = 4) and ΔIEC (n = 4) mice. Expression is normalized to *β-actin* mRNA levels. (**B**) Volcano plot displays genes whose expression was significantly upregulated (green) and downregulated (red) in RNA isolated from full thickness colonic tissue from ΔIEC mice, as compared to Ctrl mice. (**C**) RT-qPCR analysis of transcripts isolated from Ctrl (n = 4) and ΔIEC (n = 4) purified colonic epithelium. Expression is normalized to *β-actin* mRNA levels. (**D**) Representative western blot of NOS2 and TTP protein levels in mucosal scrapings from the colons of Ctrl and ΔIEC mice. (**E**) Quantification of multiple biological replicates of western blot data depicted in (**D**) (n = 3 per genotype). Levels are normalized to α-Tubulin. (**F**) Relative total nitrate/nitrite (NO_x_) levels in mucosal scrapings from the colons of Ctrl (n = 3) and ΔIEC (n = 3) mice. (*P < 0.05, ***P < 0.001). The original uncropped images of D are shown in Fig. S5.

We next focused our efforts on identifying transcripts whose expression might be regulated directly by TTP in the colonic epithelium. We purified the colonic epithelia from control and ΔIEC mice, isolated RNA, and conducted RT-qPCR analysis on a panel of characterized TTP targets including *Tnf*, *Myc*, *Vegfa*, the chemokine *Ccl2*, and others^5^. While *Tnf* and *Ccl2* levels were elevated in knockout epithelium, expression of other targets remained unchanged, suggesting that TTP may regulate expression of target genes in a tissue- or cell-specific manner (Fig. 3A). We therefore conducted RNA-deep sequencing analysis (RNA-Seq) on RNAs purified from control and knockout colons as a first step to define such targets. Overall, we identified 102 targets whose expression differed significantly (|log_2_(fold change) > 1.5|, *q*-value < 0.05, Table S1). Of these, 64 were upregulated 1.5-fold or greater and 38 were downregulated 1.5-fold or more in knockout versus control (Fig. 3B). Because TTP is a negative regulator of target transcripts, we focused on the 64 genes whose expression was upregulated in TTP knockout epithelium. To further narrow this list, we subjected them to an AU-rich element (ARE) scoring strategy which assesses the number of AREs and their proximity to one another within the 3′ UTR^22^. After applying an ARE score cutoff of greater than 4 to eliminate low probability targets, 13 of the 64 passed this threshold (Table 1). Validating the accuracy of this approach, and our screen, the canonical TTP target, *Tnf*, was amongst the top candidates and we found that TTP associates with the 3′ UTR of each of the 13 identified targets (Fig. S2A–C).

**Table 1:** Candidate target genes regulated directly by TTP in the colonic epithelium.

Gene	Fold change (log2)	P Value	Q value	ARE score	# of 3′ UTR pentamers
*Gm5615*	4.10108	5.00E-05	3.82E-03	11.15	8
*Nkx3-1*	4.09307	5.00E-05	3.82E-03	4.3	4
*Nos2*	3.44267	5.00E-05	3.82E-03	4.1	3
*Gata3*	3.05621	1.50E-04	8.64E-03	9	6
*Bmpr1b*	3.01249	5.00E-05	3.82E-03	27.85	19
*Mme*	2.57852	5.00E-05	3.82E-03	10.4	8
*Tnf*	2.07707	5.00E-05	3.82E-03	14.9	8
*Plagl1*	1.91475	5.00E-05	3.82E-03	9.5	8
*Pfkfb3*	1.7947	5.00E-05	3.82E-03	11.2	7
*Iigp1*	1.65737	1.50E-04	8.64E-03	6.2	5
*Egf*	1.60653	5.00E-05	3.82E-03	6.5	5
*Lrp8*	1.57754	1.45E-03	4.05E-02	15.7	12
*Slfn5*	1.51546	5.00E-05	3.82E-03	10.8	9

We noticed that nitric oxide synthase 2 (*iNos* or *Nos2*) was amongst the targets on this refined list of differentially expressed genes. Expression of *Nos2* in the colon must be tightly controlled in order to produce precise NO levels that promote a robust anti-microbial response without causing severe IEC cytotoxicity^17^. To determine whether *Nos2* is targeted by TTP, we first confirmed the RNA-Seq results using RT-qPCR and found that *Nos2* transcripts were elevated in purified colonic epithelium from ΔIEC mice (Fig. 3C). Likewise, we detected elevated NOS2 protein levels in the epithelium of these mice (Fig. 3D,E). NOS2 converts arginine to citrulline and produces nitric oxide (NO) as a byproduct^23^. Therefore, to determine whether elevated NOS2 in the epithelium of ΔIEC mice increased NO production, we measured the total concentration of NO metabolites (NO_x_), which includes nitrite (NO_2_-) and nitrate (NO_3_-), in mucosal scrapings from control and ΔIEC mice. Importantly, we detected elevated NO_x_ levels in ΔIEC epithelium indicating that the increases in *Nos2* transcripts and NOS2 proteins are reflected by elevated colonic NO levels (Fig. 3F).

Given that we observed increased numbers of goblet cells in ΔIEC mice (Fig. 2E) and that NOS2 and NO can promote mucus secretion and intestinal epithelial barrier integrity we determined whether epithelial deletion of TTP altered barrier function^24,25^. Indeed, the ability of paracellular probe, FITC-dextran (FD4), to translocate through the epithelial barrier and into the bloodstream was impaired in ΔIEC mice (Fig. S3). Thus, removal of TTP from IECs promotes barrier integrity, potentially through enhanced expression of *Nos2*. Taken together, these data suggest that TTP may suppress *Nos2* expression, either through direct interaction or through an indirect mechanism.

### TTP regulates *Nos2* expression through AREs embedded within its 3′ UTR

**Figure 4:**
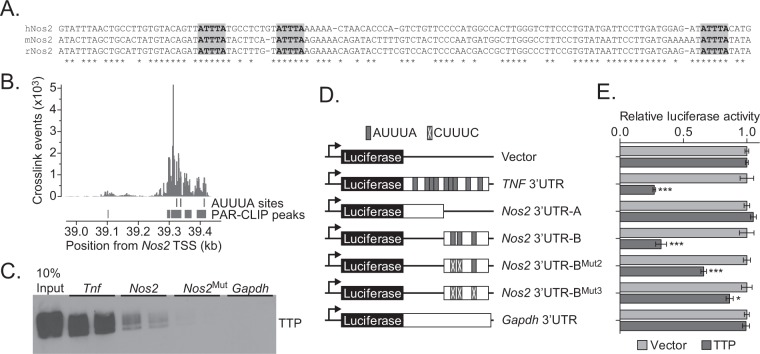
TTP regulates *Nos2* levels through AREs embedded within its 3′ UTR. (**A**) Sequence alignment of human, mouse, and rat *NOS2* 3′ UTRs. The TTP binding motifs are shaded in grey and conserved nucleotides are marked by asterisks. (**B**) PAR-CLIP binding profile for TTP on the full *Nos2* 3′ UTR in LPS stimulated macrophages. This data was obtained from the TTP Atlas^26^. TTP binding motifs and PAR-CLIP peaks are indicated below the graph. (**C**) Biotinylated RNA pulldown using the 3′ UTRs of the indicated transcripts as probes. The *Nos2*^Mut^ probe contained mutations that changed the three AUUUA pentamers to CUUUC. Two replicate experiments are shown for each probe. (**D**) Schematic of luciferase 3′ UTR reporter constructs used in (**E**). TTP binding motifs are indicated by grey boxes, which contain an X if that motif has been mutated to CUUUC. (**E**) Luciferase assays using the indicated reporters in vector or TTP transfected HEK293 cells. Error bars represent SEM. (*P < 0.05, ***P < 0.001). The original uncropped images of C are shown in Fig. S6.

The downstream portion of the human *NOS2* 3′ UTR contains three AREs that are conserved in rat and mouse (Fig. 4A). To determine whether TTP binds *Nos2*, we first searched the TTP Binding Atlas, which contains datasets of transcripts that directly interact with TTP as identified through the Photoactivatable Ribonucleoside-Enhanced Crosslinking and Immunoprecipitation (PAR-CLIP) technique^26^. We uncovered evidence for robust TTP binding to the *Nos2* 3′ UTR in reported data from macrophages that were stimulated by lipopolysaccharide (LPS) treatment (Fig. 4B)^26^. To determine whether TTP associates with the *Nos2* 3′ UTR, we performed a biotinylated oligonucleotide pull-down assay. Probes designed to encompass the three AREs within the *Nos2* 3′ UTR were incubated with protein lysate from HEK293 cells that were first transfected with a plasmid harboring a cDNA encoding TTP. The complexes were precipitated with streptavidin conjugated magnetic beads, and eluted TTP was detected by western blot. Probes designed against the full *TNF* and *Gapdh* 3′ UTRs were included as positive and negative controls, respectively. We found that the *Nos2* 3′ UTR precipitated TTP, albeit not as strongly as the *TNF* 3′ UTR (Fig. 4C). Furthermore, the TTP-*Nos2* interaction was ablated when the AREs in the *Nos2* 3′ UTR were mutated (Fig. 4C).

To determine whether TTP binding to the *Nos2* 3′ UTR stimulates transcript degradation, we employed a luciferase stability assay. We subcloned fragments of the *Nos2* 3′ UTR, as well as the 3′ UTRs of *TNF* and *Gapdh* as controls, downstream from the luciferase gene in the pmirGLO vector backbone (Fig. 4D). We found that the downstream portion of the *Nos2* 3′ UTR reduced luciferase levels in TTP transfected cells (Fig. 4E). This reduction was nearly fully reversed when all three AREs were mutated (Fig. 4E). Thus, TTP binds the *Nos2* 3′ UTR and reduces levels of luciferase transcripts when fused downstream. Together, these results suggest that TTP targets *Nos2* through the AREs within its 3′ UTR.

### LPS induces TTP expression in IECs

**Figure 5:**
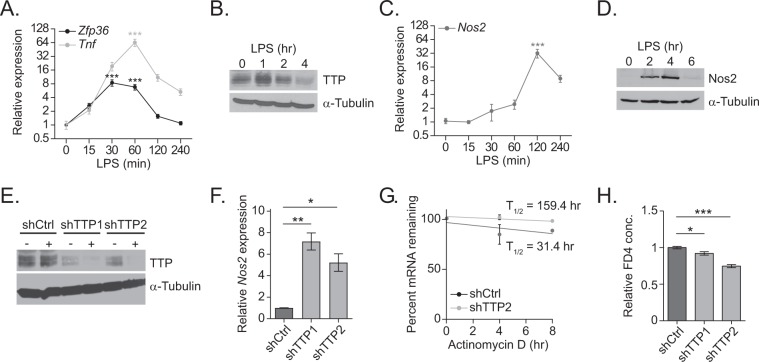
LPS induces TTP expression in IECs. (**A**) RT-qPCR analysis of *Tnf* and *Zfp36* transcripts in RNA isolated from rIEC6 cells stimulated with LPS for the indicated times. Expression levels are normalized to *β-actin*. (**B**) Western blot analysis of TTP protein levels in rIEC6 cells stimulated with LPS for the indicated times. (**C**) RT-qPCR analysis of *Nos2* transcripts in RNA isolated from rIEC6 cells stimulated with LPS for the indicated times. Expression levels are normalized to *β-actin*. (**D**) Western blot analysis of NOS2 protein levels in rIEC6 cells stimulated with LPS for the indicated times. (**E**) Western blot analysis of TTP protein levels in TTP-silenced rIEC6 cells. Ctrl- and TTP-specific shRNAs were induced by DOX for 72 hrs. (**F**) RT-qPCR analysis of transcripts isolated from TTP-silenced IEC6 cells after induction with LPS for 2 hours. Expression levels are normalized to *β-actin* mRNA levels. (**G**) Control and TTP-silenced IEC6 cells were transfected with the pmirGLO *Nos2* 3′ UTR-B construct. *Nos2* 3′ UTR-regulated firefly luciferase mRNA stability was assessed by RT-qPCR after treatment with actinomycin D for the indicated times. Expression levels are normalized to renilla luciferase mRNA levels. (**H**) FD4 permeability analysis of monolayers of rIEC6 cells with the indicated shRNA. Error bars represent SEM. (*P < 0.05, **P < 0.01, ***P < 0.001). The original uncropped images for B, D and E are shown in Fig. S7.

IECs express toll-like receptor four (TLR4), which is a pattern recognition receptor that upon binding LPS released from the cell wall of gram (-) bacteria, stimulates expression of pro-inflammatory cytokines including *Tnf* ^27,28^. While this pathway stimulates *Zfp36* expression in macrophages, it is unknown whether this is also true in IECs^29^. To test this hypothesis, we stimulated the rat intestinal epithelial cell line, rIEC6, with LPS over a period of four hours, harvested cells at various time-points, and quantified *Zfp36* expression. We also monitored *Tnf* induction, whose expression level reached maximal levels at 1 hr of treatment, indicating that the TLR4-signaling pathway was operational in these cells (Fig. 5A). *Zfp36* transcripts were induced 8-fold at the 30-minute time-point and then decreased to base-line levels by four hours (Fig. 5A). This rapid decay in *Zfp36* transcripts is consistent with the fact that TTP directly regulates the *Zfp36* transcript itself ^30–32^. TTP protein levels were induced at one hour of treatment and then return to levels below baseline at four hours (Fig. 5B). We next monitored *Nos2* levels and found that LPS treatment for 2 hours induced *Nos2* expression 32-fold (Fig. 5C). Moreover, while there was no detectable NOS2 protein in untreated cells, LPS stimulated NOS2 protein levels at the two- and four-hour time points (Fig. 5D). Importantly, NOS2 levels were nearly absent in cells treated with LPS for six hours, indicating negative regulators tightly control its expression (Fig. 5D).

To test whether TTP negatively regulates *Nos2* transcripts in rIEC6 cells, we created cell lines that express two shRNAs that target distinct regions of the *Zfp36* transcript and a third expressing a scrambled shRNA sequence as a control. These shRNAs (denoted as shTTP1 and  shTTP2) were placed under regulation of a doxycycline (Dox) inducible promoter. After treating cells with Dox for 72 hours, very little TTP proteins were detected (Fig. 5E). Next, we treated these cells with LPS for two hours and found that TTP-depletion led to a dramatic increase in *Nos2* transcript levels (Fig. 5F). We next determined whether TTP suppressed *Nos2* expression through its 3′ UTR by destabilizing the transcript. To test this mechanism, we transfected the *Nos2* 3′ UTR luciferase reporter plasmid into control and TTP-depleted IEC6 cells, inhibited transcription using actinomycin D, and measured the decay of the *luciferase*-*Nos2* 3′UTR fusion transcript. Depletion of TTP resulted in an approximately 5-fold increase in the half-life of this fusion transcript compared to control IEC6 cells (Fig. 5G).

Since we observed enhanced epithelial barrier integrity in ΔIEC mice, we examined whether this was an epithelial cell intrinsic phenomenon. Indeed, depletion of TTP from rIEC6 cells prohibited the paracellular translocation of FD4 through rIEC6 cells grown as a monolayer on transwell supports (Fig. 5H). These findings suggest that TTP participates in a negative feedback loop to control *Nos2* expression in LPS-stimulated rIEC6 cells.

### ΔIEC mice are less susceptible to DSS-induced colitis

**Figure 6:**
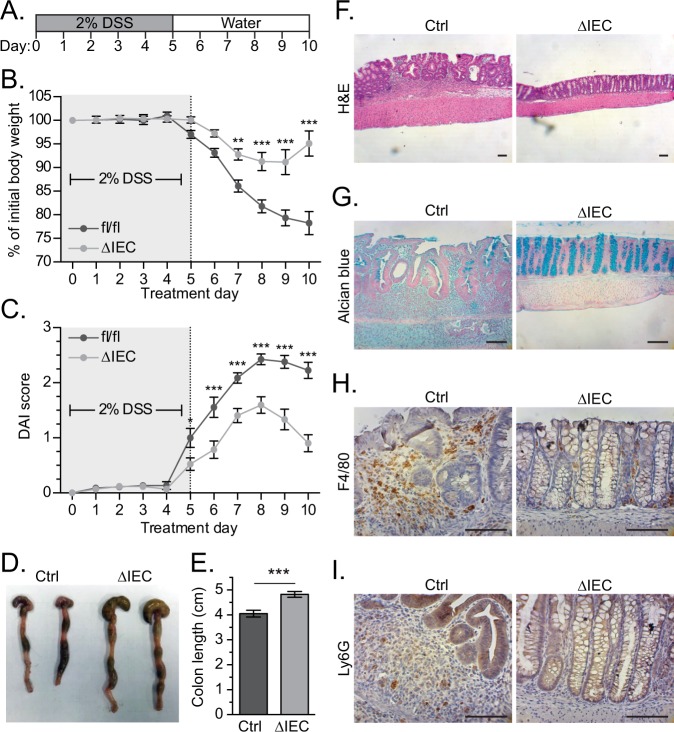
ΔIEC mice are less susceptible to DSS-induced colitis. (**A**) Schematic of the DSS protocol, where 2% DSS was administered to 6-8-week-old female mice in the drinking water for 5 days, after which it was replaced with normal water for an additional 5 days. (**B**) Weight measurements of mice (Ctrl, n = 15; ΔIEC, n = 14) throughout the DSS protocol. (**C**) As in **B**, except disease activity indices (DAIs) were assessed. (**D**) Representative image of colons from Ctrl and ΔIEC mice at day 10. (**E**) Quantification of colon lengths at day 10 (Ctrl, n = 15; ΔIEC, n = 14). (**F**) H&E, (**G**) alcian blue, (**H**) F4/80, and (**I**) Ly6G staining of colonic sections from Ctrl and ΔIEC on day 10. Error bars represent SEM (*P < 0.05, **P < 0.01, ***P < 0.001).

Ulcerative colitis (UC) is a chronic inflammatory condition affecting the large bowel^33^. Recently it was demonstrated that while NO derived from macrophages aggravated colitis, NO produced by IECs promoted recovery in a mouse model of UC^34^. A common model of acute colitis in mice involves supplying dextran sodium sulfate (DSS) in the drinking water which compromises the epithelial barrier and allows infiltration of luminal microbes^35^. Penetrating microbes elicit an immune response and IECs respond by regenerating the mucosal barrier^36^. Given our results that TTP negatively regulates *Nos2*, we predicted that ΔIEC mice may display an altered response to DSS colitis. To investigate this possibility, we supplied control and ΔIEC mice with 2% DSS in their drinking water for five days, after which we provided normal drinking water for five days to facilitate recovery (Fig. 6A). Whereas control mice steadily lost weight during the recovery period, ΔIEC mice initially lost weight and then began to recover during the latter stages of the protocol (Fig. 6B). We also monitored the disease activity index (DAI), which is a scoring system that accounts for weight loss, rectal bleeding, and stool consistency^37^. In comparison to controls, ΔIEC mice displayed significantly improved DAI scores from days five to ten (Fig. 6C). Shortening of the colon occurs in humans with UC and this phenotype accompanies acute DSS colitis in mice^38,39^. At the end of the protocol, colons were harvested and measured. The colons of ΔIEC mice were significantly longer than control mice consistent with observations that TTP deletion lessens the severity of DSS colitis (Fig. 6D,E).

There are distinct histological changes in the colons of UC patients that are also seen in mouse models of colitis including crypt distortion, inflammation, increased bowel wall thickness, and loss of goblet cells^33,39^. Using IHC, we assessed these parameters in sections prepared from control and ΔIEC mice harvested at the end of the DSS-colitis protocol. Hematoxylin and eosin staining of sectioned colons revealed a largely normal colonic architecture in ΔIEC mice, whereas control mice exhibited residual crypt distortion and ulceration, pronounced infiltration of immune cells, and increased bowel wall thickness (Fig. 6F). Additionally, through Alcian blue staining, we observed that whereas goblet cells failed to repopulate control mice, they re-occupied their expected position along the crypt axes in knockout mice (Fig. 6G). Finally, we assessed the immune cell infiltration in the colon tissue at the end of the DSS-colitis. We identified fewer macrophages and neutrophils in ΔIEC mice by staining with anti-F4/80 and anti-Ly6G antibodies, respectively (Fig. 6H,I). Thus, loss of TTP expression in intestinal epithelial cells lessens the severity of acute colitis.

## Discussion

Germline *Zfp36* knockout mice (*Zfp36*^−/−^) fail to thrive due to systemic inflammation, demonstrating the critical role of TTP in immune-response resolution^9^. A subsequent study demonstrated this phenotype was not fully recapitulated upon removal of TTP from myeloid cells, suggesting that a compendium of cell types require the immunomodulatory function of TTP^19^. Indeed, an immunoregulatory role for TTP has been described in keratinocyte-specific *Zfp36* knockout mice and mammary epithelium-specific *Zfp36* knockout mice^40,41^. However, this does not seem to be the primary role for TTP in all cell types. Indeed, Wang *et al*., reported that TTP expression in CD8^+^ T-cells instead restricts their proliferation^42^. Here, we also report IEC-specific *Zfp36* deletion leads to expansion of the proliferative zone and increased goblet cell numbers in the colonic crypts. Therefore, it is critical to continue defining the cell-type specific functions of TTP.

Here we demonstrate that TTP controls *Nos2* expression during intestinal homeostasis. *Nos2* is regulated extensively at the post-transcriptional level by RNA-binding proteins (RBP) and microRNAs^43^. Fechir *et al*., were the first to report a role for TTP in *Nos2* post-transcriptional regulation^44^. However, in their study, recombinant TTP was unable to directly bind to the human *NOS2* 3′ UTR in a UV crosslinking assay^44^. A later report from this group demonstrated that TTP stabilized the *NOS2* transcript by sequestering another RBP, KSRP, which directly binds and destabilizes *NOS2*^45^. Here we demonstrate that TTP is able to interact with the mouse *Nos2* 3′ UTR through three AREs. Furthermore, we found that TTP can suppress expression of a reporter gene containing the *Nos2* 3′ UTR and this depends largely on the presence of the three AREs. Because TTP has been predominantly shown to destabilize transcripts, we favor a model where TTP directly binds the *Nos2* 3′ UTR in intestinal epithelial cells. However, we cannot exclude the possibility that TTP is being tethered to *Nos2* by another ARE-binding protein. This also raises the possibility that post-transcriptional gene regulation is finely tuned in distinct cellular contexts through post-translational modification and precise coordination of a multitude of RNA-binding proteins and non-coding regulatory RNAs. Indeed, TTP and KSRP have also been shown to work in concert to destabilize the *IL-8* 3′ UTR^46^. Also, when comparing the transcriptomes of macrophages and neutrophils derived from *Zfp36*^−/−^ mice, Ebner *et al*., found that *Nos2* levels were only dysregulated in neutrophils^47^. The combination of a diverse set of RBPs, microRNAs, and RBP-modifying enzymes in each cell may dictate how each TTP target is regulated.

TTP has been shown to have important functions in many disease models, including endotoxin shock, psoriasis, and diabetes^19,40,48^. In this study, we investigate the role of TTP in a model of colitis. We see that, surprisingly, loss of TTP from IECs did not aggravate the colitis-associated inflammation, but instead protected mice from colitis symptoms. This surprising result may be due to pleiotropic effects of TTP on several aspects of IEC biology. Indeed, three phenotypes observed in ΔIEC mice may contribute to a decreased colitis severity. First, we observed enhanced epithelial cell proliferation in ΔIEC mice, which could promote IEC regeneration after DSS-induced colitis. Second, we observed increased goblet cells numbers in ΔIEC mice, which could protect the epithelium from an insult or promote restitution of the mucosal barrier after an insult. Finally, we observed reduced epithelial barrier permeability in ΔIEC mice, which could diminish the effects of DSS on the epithelium and prevent translocation of microorganisms from the colonic lumen to the underlying tissue. While *Nos2* may contribute to these phenotypes or provide an additional defense against severe DSS colitis, we cannot eliminate the possibility that other TTP target genes also contribute.

The role for NOS2 in intestinal disease has been controversial. There are studies demonstrating that NOS2 expression and NO production both aggravates and protects against pathologies associated with acute colitis^16–18,49^. There are at least two possible explanations for this discrepancy. First, the precise level of NOS2 expression is likely critical. NO production is essential in the intestines as it promotes mucus secretion^24,50^, epithelial cell migration^51^, barrier integrity^52^, and microbial cytotoxicity during bacterial infection^13,15^, while suppressing the function of resident immune cells^16^. However, high NO levels can induce IEC apoptosis, thus causing epithelial barrier dysfunction^53^. Some NO metabolites can also mutagenize the DNA of IECs, thus restriction of NO levels is critical to prevent tumorigenesis^54^. Here we show that TTP loss from IECs causes only a 2-fold increase in mucosal NO_x_ levels, which is likely not high enough to be detrimental to the IECs. Second, the cell from which NOS2 is expressed contributes to its role in disease. Indeed, Stettner *et al*., demonstrated that increasing NO metabolism in IECs promotes recovery from colitis by reducing macrophage infiltration and tissue damage, yet NO production in macrophages leads to their activation and exacerbation of the disease^34^. We also show that mice producing NOS2 in IECs fare better in a model of experimental colitis. This may also explain why Joe *et al*., found that germline *Zfp36*^−/−^ mice are slightly more susceptible to induced colitis^55^. In that study, while TTP is deleted from the IECs of *Zfp36*^−/−^ mice, TTP is also lost in the macrophages, leading to elevation of *Nos2* and other cytokines in these invading cells^55^. Therefore, the beneficial effect of deleting TTP from IECs is offset by the detrimental effect of macrophage-induced inflammation.

In conclusion, our study is the first to ascribe a function to TTP in normal IECs. We show that TTP suppresses IEC proliferation, goblet cell numbers, and *Nos2* expression. We also demonstrate that *Nos2* is targeted by TTP through AREs in its 3′ UTR. TTP exacerbates the development of colitis, potentially through the suppression of *Nos2*. These findings raise interesting questions about the cell specific roles of both TTP and NOS2 in intestinal homeostasis and colitis. Stabilization of TTP is a promising therapeutic avenue in a variety of inflammatory conditions, yet our findings suggest that this approach should be carefully considered in a tissue-specific manner as it may be detrimental in some diseases, such as IBD^56,57^. Our results affirm the need for sustained research into the function of this pleotropic RNA-binding protein in additional cell types to fully understand its role in homeostasis and disease.

## Materials and Methods

### Mice

*Villin:Cre* mice were obtained from Jackson Laboratories and *Zfp36*^fl/fl^ mice have been described previously^19^. Intestinal epithelial cell-specific TTP knockout mice, termed ΔIEC, were generated by crossing *Zfp36*^fl/fl^ mice with *Villin:Cre* mice expressing CRE recombinase under the control of the *Villin* promoter. All experiments were performed using *Zfp36*^fl/fl^ littermates as controls. Mice were routinely genotyped by isolating genomic DNA from a 2 mm tail biopsy as described previously^58^. The floxed *Zfp36* allele was amplified as described previously and the *Cre* transgene was amplified as recommended by Jackson Laboratories using the indicated primers (Table S2)^19^. Tissue-specific knockout was confirmed by first using the MyTaq Extract-PCR Kit (Bioline) to isolate genomic DNA from 2 cm of colon tissue. Subsequently, multiplexed PCR was used to amplify either the floxed or deleted *Zfp36* alleles with the oligonucleotides described previously and listed in Table S2^19^. Unless otherwise indicated, all experiments were performed on 6-8-week-old mice. All animal breeding and experiments were approved by the Institutional Animal Care and Use Committee (IACUC) at the Pennsylvania State University College of Medicine. All experiments were performed in accordance with relevant guidelines and regulations provided by the IACUC.

### Cell culture

rIEC6 (ATCC) and HEK293FT (Invitrogen) cell lines were maintained at 37 °C in 5% CO_2_ in DMEM supplemented with 10% FetalPlex (Gemini), 50 units/mL penicillin (Corning Mediatech), 0.1 mg/mL streptomycin (Corning Mediatech), and 2 mM Glutamax (ThermoFisher Scientific). HEK293FT cells were supplemented with 500 μg/mL G418 (VWR).

### RNA isolation and RT-qPCR

To assess gene expression in mouse colons, total RNA was isolated by pestle homogenization of 2 cm of tissue in TRIzol according manufacturer’s instruction. In rIEC6 cells, total RNA was isolated after treatment with 1 μg/mL LPS (Sigma) for the indicated times. The iScript cDNA synthesis kit (Bio-Rad, 170-8891) was used to synthesize cDNAs, in which gene expression was measured by quantitative PCR using the indicated primers (Table S2) and the SensiFAST kit (Bioline, Bio-96020) as described previously^59^. The data is presented as relative expression (2^−ΔΔCt^) with *β-actin* serving to normalize expression values.

### Epithelial cell purification

Purified IECs were isolated from control and ΔIEC mice as described previously^60^. Briefly, colons were opened longitudinally, washed thoroughly with PBS, and cut into 5 mm fragments. IECs were separated from stromal cells by incubation in cold chelation buffer (5.6 mM Na2HPO4, 8 mM KH2PO4, 96.2 mM NaCl, 1.6 mM KCl, 43.4 mM sucrose, 54.9 mM d-sorbitol, 0.5 mM dithiothreitol [DTT]) containing 2 mM EDTA, followed by mechanical disruption through repeated pipetting of the tissue fragments. IECs were collected and filtered through a 70 μm filter then pelleted by centrifugation at 850 × g for 5 min. Cells were resuspended in TRIzol and RNA was isolated as described above.

### Western blot

To assess protein expression in mouse tissue, 2 cm of distal colon was suspended in RIPA buffer supplemented with protease inhibitors (10 μg/ml leupeptin, 10 μg/ml aprotinin, and 1 mM phenylmethylsulfonyl fluoride) and homogenized using a pestle homogenizer. Mucosal scrapings were prepared from entire mouse colons after vigorous washing with PBS and were subsequently resuspended in RIPA buffer with protease inhibitors. Approximately 1 × 10^6^ rIEC6 cells were plated in a 10 cm dish, treated the following day with 1 μg/mL LPS for the indicated times, pelleted, and resuspended in RIPA buffer. Protein extracts were prepared as described previously and analyzed using western blot with the following antibodies and dilutions: anti-TTP (Millipore, ABE283, 1:500), anti-NOS2 (Abcam, ab178945, 1:1000), and anti-α-tubulin (Sigma T9026, 1:1000)^61^.

### Histology and immunohistochemistry

Sections of colonic tissue were fixed in formalin overnight at room temperature and then transferred to 70% EtOH and stored at 4 °C until they were embedded in paraffin. For staining, slides were dewaxed with xylene and rehydrated by serial alcohol washes. For alcian blue staining, slides were incubated in 1% alcian blue in 3% acetic acid for 4 minutes and counterstained with nuclear fast red. For alkaline phosphatase staining, the Vector Laboratories BCIP/NBT AP substrate kit (SK-5400) was used according to the manufacturer’s instructions. For immunohistochemistry, antigen retrieval was performed by incubating slides in TE buffer (pH 9) for 20 minutes in a standard rice cooker. After cooling, slides were peroxidase quenched and blocked in 10% normal goat serum (Abcam). The slides were incubated overnight at 4 °C with the following primary antibodies: anti-Ki67 (Vector Laboratories, VP-RM04, 1:400), anti-lysozyme (Abcam, ab108508, 1:1000), anti-chromogranin A (Abcam, ab15160, 1:800), anti-F4/80 (Santa Cruz, sc-59171, 1:400), and anti-Ly6G (Biolegend, 1A8, 1:400). The following day, slides were incubated with either goat anti-rabbit or goat anti-rat secondary antibody (Vector Laboratories) for 1 hour at room temperature, detected with ABC and DAB kits (Vector Laboratories), and counterstained with hematoxylin. Periodic acid-Schiff (PAS) and Hematoxylin and eosin (H&E) staining was performed by the Morphological and Molecular Pathology Core Research Lab at the Penn State University College of Medicine.

### RNA-sequencing

Total RNA was isolated from 2 cm of full thickness colonic tissue by pestle homogenization in TRIzol according to the manufacturer’s instructions. RNA concentrations were measured on a NanoDrop (ThermoFisher) apparatus then synthesized into cDNA libraries using the NEXTflex Rapid Directional RNA Sequencing Kit (BioO Scientific). The libraries underwent quality control for quantity and fragment length size distribution then were sequenced using a HiSeq. 2500 according to the manufacturer’s instructions. Sequence read were trimmed and filtered using the FASTX-Toolkit then aligned to the mouse reference (mm10) using Tophat v2.0.9. Fragments Per Kilobase Of Exon Per Million Fragments Mapped (FPKM) values were calculated using Cufflinks v2.2.1 and differential expression analysis was done using CuffDiff. The RNA-Seq data was deposited on the Gene Expression Omnibus database and can be accessed using reference number, GSE123345.

### Nitrate/nitrite measurements

Colons were excised, opened longitudinally, and washed with PBS. Mucosal scrapings were prepared using a microscope coverslip and subsequent homogenization in PBS using a pestle homogenizer. The samples were clarified by centrifugation at 10,000 × g for 20 minutes. Nitrate in the supernatant was converted to nitrite and measured using the Nitrate/Nitrite Colorimetric Assay Kit (Cayman Chemicals) following the manufacturer’s instructions.

### Intestinal permeability measurements

Control and ΔIEC mice were administered 44 mg/100 g body weight 4 kDa fluorescein isothiocyanate (FITC)-dextran (Sigma) by oral gavage as previously described^62^. After 4 hours, mice were euthanized, and blood samples were rapidly collected using cardiac puncture. Blood was allowed to clot for 30 minutes at room temperature in the dark, then serum was isolated by centrifugation. Serum was diluted 1:2 in PBS and the FITC-dextran concentration was measured using a fluorometer (BioTek, Synergy 2) (excitation wavelength, 485 nm; emission wavelength, 528 nm). For *in vitro* assessment of barrier function, approximately 1 × 10^4^ rIEC6 cells were seeded onto tissue culture treated 6.5 mm Costar transwell permeable supports in a 24 well plate. Cells were grown to confluence over 6 days in DMEM containing 1 μg/mL doxycycline to activate the shRNAs. Subsequently, the media in the upper chamber was replaced with 100 µL of DMEM supplemented with 1 μg/mL FD4. The plate was incubated for two hours at 37 °C, after which the FITC-dextran concentration in the lower chamber was measured using a fluorometer (BioTek, Synergy 2) (excitation wavelength, 485 nm; emission wavelength, 528 nm).

### Plasmids

SMARTvector inducible lentiviral shRNA constructs were obtained from Dharmacon. The sequences and clone numbers are listed in Table S2. Construction of the pcDNA3-TTP vector was described previously^63^. For the luciferase reporter constructs, pmirGLO (Promega) was used as the vector backbone. The *TNF* 3′ UTR was amplified from human genomic DNA and the *Nos2* and *Gapdh* 3′ UTRs were amplified from mouse genomic DNA using the indicated primers (Table S2). The PCR products were subcloned into pmirGLO as SacI-SalI fragments. ARE mutants were generated using the Q5 Site-Directed Mutagenesis Kit (NEB) and the indicated primers (Table S2). All inserts were verified by Sanger sequencing.

### Biotin pulldown

Biotin pulldowns were performed as previously described^63^. Briefly, biotinylated probes were generated by amplifying the *TNF*, *Nos2*, and *Gapdh* 3′ UTRs from the respective pmirGLO plasmid using forward primers that contained a T7 RNA polymerase promoter sequence (Table S2). From this amplified DNA, biotinylated mRNAs were synthesized using the MAXIscript *in vitro* transcription kit (Ambion) per the manufacturer’s instructions, using a ratio of 1:5 biotin-labeled CTP to unlabeled CTP. HEK293 cells were transfected with 5 μg of pcDNA3-TTP using lipofectamine. After 24 hours, cells were lysed with PLB supplemented with protease inhibitors (10 μg/ml leupeptin, 10 μg/ml aprotinin, and 1 mM phenylmethylsulfonyl fluoride). Lysates were cleared by centrifugation at 16,000 × g. Reactions containing 500 nM of biotinylated transcripts were mixed with 40 μg of TTP-transfected HEK293 cell lysate for 30 min at room temperature, then incubated with 15 μL streptavidin-dynabeads (Invitrogen) for 30 min, with periodic disruption for brief vortexing. The beads were washed twice with PBS with 500 mM NaCl, proteins were eluted by boiling in 1X SDS-PAGE loading buffer, and samples were separated by SDS-PAGE and analyzed by western blot using anti-TTP antibody (Millipore, ABE283, 1:500).

### Luciferase assay

HEK293 cells were seeded on 24-well plates in quadruplicate. The following day, cells were transfected using Lipofectamine 2000 (Invitrogen). Each transfection contained 100 ng of the pmirGLO 3′ UTR reporter plasmid, which also expresses Renilla luciferase from a constitutive promoter, 50 ng of either pcDNA3 or pcDNA3-TTP expression plasmid, and 350 ng pBluescript. The next day, cells were lysed in 100 μl of 1 × passive lysis buffer (Promega), and luciferase levels were measured using a Firefly & Renilla Luciferase Single Tube Assay Kit (Biotium, 30081) on a GloMax 20/20 luminometer (Promega). Firefly luciferase levels were normalized to renilla luciferase levels to control for transfection efficiency.

### Generation of stable rIEC6 cell lines

Lentiviral particles were produced by transfecting HEK293FT cells with packaging vectors, pMD2 (2.8 μg) and psPAX2 (5.2 μg), and 5 μg of the respective SMARTvector shRNA construct using Lipofectamine 2000 (Life Technologies). Media containing virus was harvested at 48 and 72 h following transfection and cleared of cell debris by centrifugation at 1500 × g for 5 min. The media was supplemented with 6 μg/mL hexadimethrine bromide (Sigma) and added to rIEC6 cells for 10 h on subsequent days. The media containing virus was replaced with fresh DMEM after each infection. Media was supplemented with 3 μg/mL puromycin (Sigma) 24 hours after the second infection, and cells were selected for 1 week. Stable pools were maintained with 1 μg/mL puromycin. Control and *Zfp36*-specific shRNAs were induced in rIEC6 cells with 1 μg/mL doxycycline (Acros Organics) for 72 hours prior to experimental analysis.

### Measurement of RNA stability

IEC6 cells were seeded on 60 mm dishes. The following day, control and *Zfp36*-specific shRNAs were induced in rIEC6 cells with 1 μg/mL doxycycline (Acros Organics). After 24 hours cells were transfected with 4 μg of the pmirGLO *Nos2* 3′ UTR-B reporter plasmid and 4 μg pBluescript. The next day, cells were treated with 5 μg/mL Actinomycin D (MP Biomedicals) for 0, 4, or 8 hours. Subsequently, total RNA was isolated and converted to cDNA, as described above, and firefly luciferase transcript levels were measured by quantitative PCR using the indicated primers (Table S2). The data is presented as percent mRNA remaining with renilla luciferase expression serving to normalize values.

### DSS colitis

Acute colitis was induced by administering 2% DSS (TdB Consulting, DB001) to 6-8-week-old female control (*Zfp36*^fl/fl^) and ΔIEC mice in their drinking water for 5 days. Afterwards, the mice were provided normal drinking water and allowed to recover for 5 additional days. Throughout the protocol, mice were weighed daily and disease activity indices (DAIs) were assessed on a scale from 0 to 4, with 4 being the most severe as described previously^37^. This score was based on body weight loss, stool consistency, and rectal bleeding and is presented as an average score of these parameters each day. At the end of the protocol, colon length was measured, and distal colonic tissue samples were collected, fixed in formaldehyde, and paraffin-embedded.

### Statistical analysis

Values are presented as mean ± standard error of the mean, with the number of independent replicates indicated by *n*. Statistical comparisons between groups was done using two-tailed, unpaired student’s t-tests. Differences in weight and DAI score over the course of the DSS protocol were compared using a 2-way ANOVA with multiple comparisons.

## Supplementary information

Supplementary information

## Data Availability

All data generated or analyzed during this study are included in this published article and its Supplementary Information Files.
