# Supplementary material for: Tristetraprolin targets Nos2 expression in the colonic epithelium
Source: Sci Rep. 2019 Oct 8;9:14413. doi: 10.1038/s41598-019-50957-9 (PMC6783411; doi:10.1038/s41598-019-50957-9)
Supplement: Supplementary file 1 — Supplementary information [file 41598_2019_50957_MOESM1_ESM.pdf]

Supplementary Information

Tristetraprolin targets *Nos2* expression in the colonic epithelium

Melanie A. Eshelman[1], Stephen M. Matthews[1,2], Emily M. Schleicher[1], Rebecca M. Fleeman[1], Yuka Imamura Kawasawa[3,4], Deborah J. Stumpo[5], Perry J. Blackshear[5,6], Walter A. Koltun[2], Faoud T. Ishmael[7], Gregory S. Yochum[1,2,*]

[1]Department of Biochemistry & Molecular Biology, Pennsylvania State University College of Medicine, Hershey, PA, 17033, USA.

[2]Department of Surgery, Division of Colon & Rectal Surgery, Pennsylvania State University College of Medicine, Hershey, PA, 17033, USA.

[3]The Institute for Personalized Medicine, Pennsylvania State University College of Medicine, Hershey, PA 17033, USA.

[4]Department of Pharmacology, Pennsylvania State University College of Medicine, Hershey, PA, 17033, USA.

[5]Signal Transduction Laboratory, National Institute of Environmental Health Sciences, Research Triangle Park, NC, 27709, USA.

[6]Departments of Medicine & Biochemistry, Duke University Medical Center, Durham, NC, 27710, USA.

[7]Department of Allergy & Sleep Medicine, Mount Nittany Medical Group, State College, PA, 16803, USA.

[*]Correspondence and requests for materials should be addressed to Gregory S. Yochum (e-mail: gsy3@psu.edu)

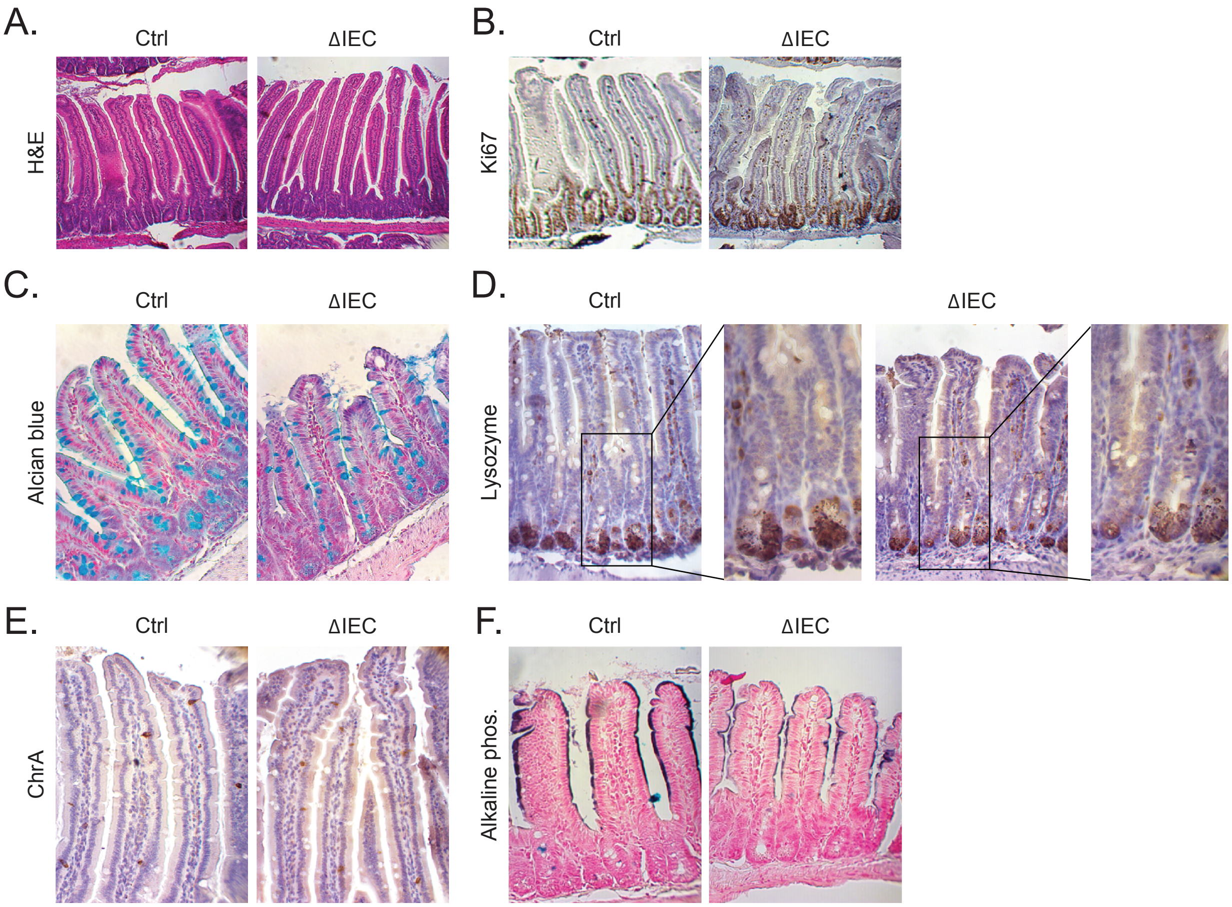

Figure S1. TTP loss does not severly compromise the architecture or cellular composition of the small intestine. (**A**) H&E, (**B**) Ki67, (**C**) alcian blue, (**D**) lysozyme, (**E**) chromogranin A, and (**F**) alkaline phosphatase staining of small intestine sections from 6-week-old control (Ctrl.) and ΔIEC mice.

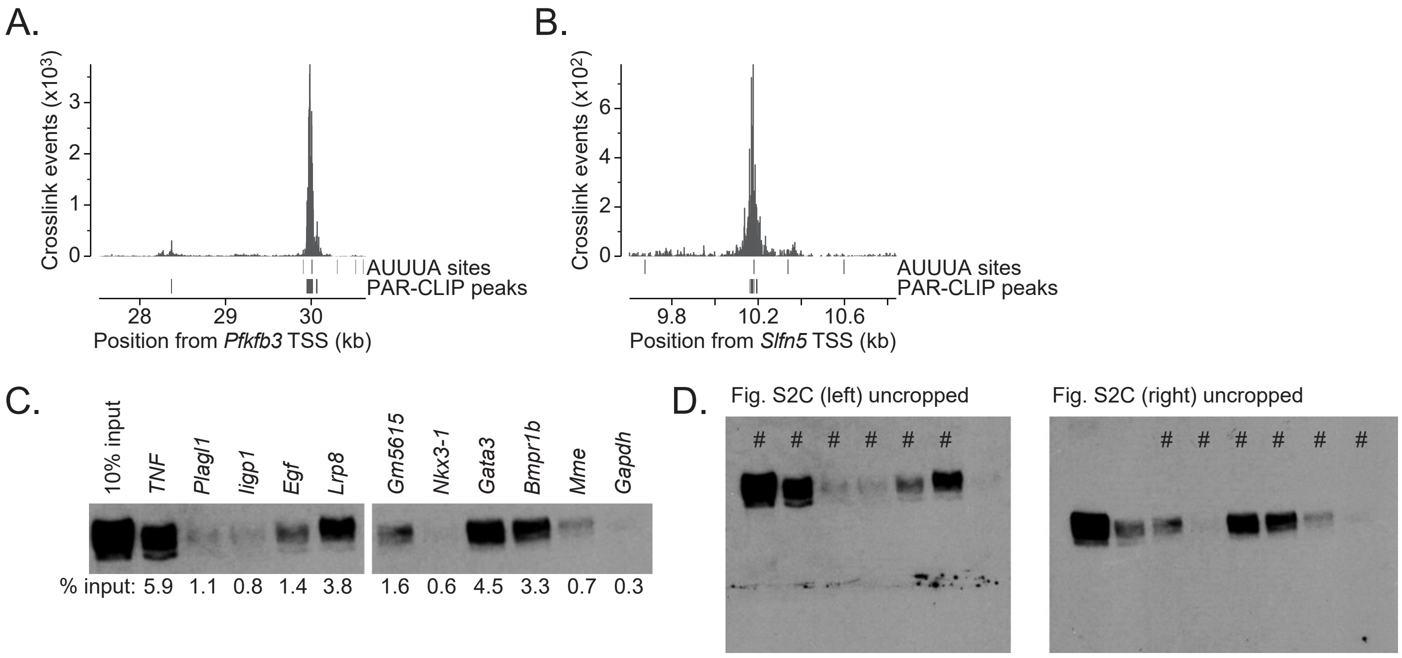

Figure S2. (**A** and **B**) PAR-CLIP binding profiles for TTP on the 3'UTRs from the indicated transcripts in LPS stimulated macrophages. This data was obtained from the TTP Atlas[26]. TTP binding motifs and PAR-CLIP peaks are indicated below each graph. (**C**) Biotinylated RNA pulldown using the 3' UTRs of the indicated transcripts as probes. (**D**) Uncropped images for **C**. # indicates the lanes containing the cropped bands shown in Fig. S2C.

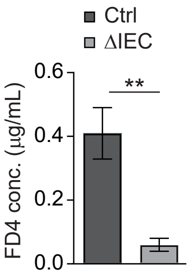

Figure S3. Concentration of FD4 in the serum of Ctrl (n=4) and ΔIEC (n=4) mice 4 hours after oral gavage of FD4. Error bars represent SEM. (**P < 0.01).

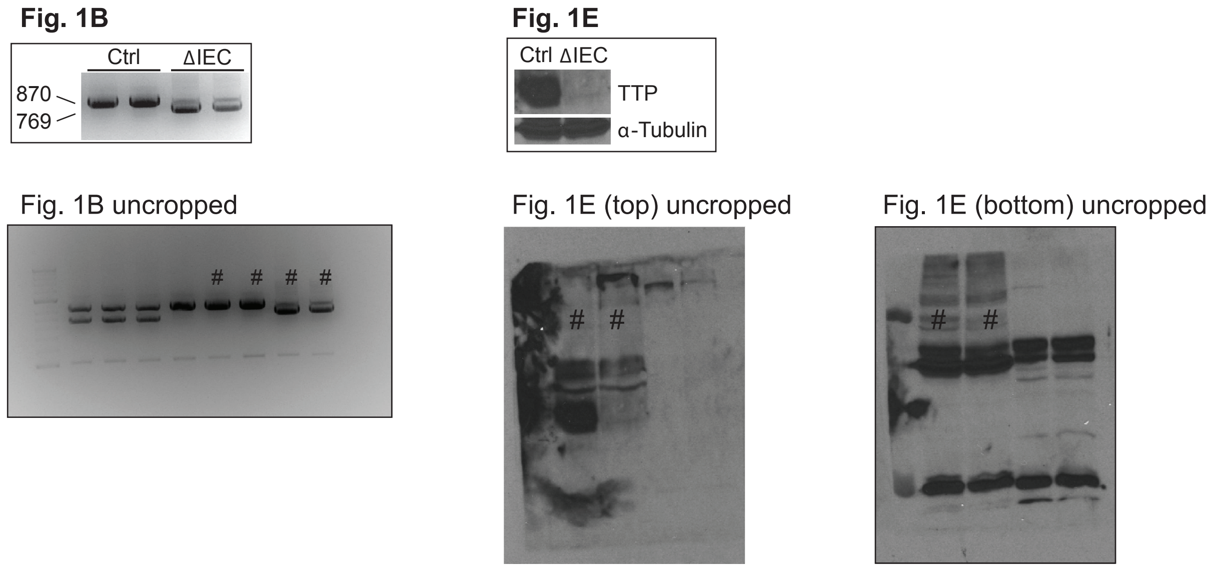

Figure S4. Fig. 1B, 1E and the original uncropped images. # indicates the lanes containing the cropped bands shown in Fig. 1B and 1E.

Fig. 3D

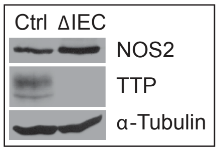

Fig. 3D (top) uncropped

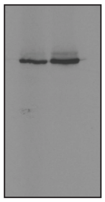

Fig. 3D (middle) uncropped

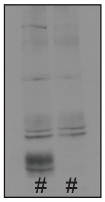

Fig. 3D (bottom) uncropped

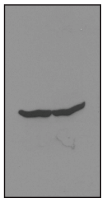

Figure S5. Fig. 3D, and the original uncropped images. # indicates the lanes containing the cropped bands shown in Fig. 3D (middle).

Fig. 4C

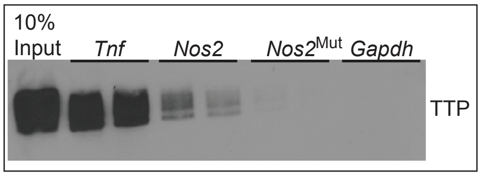

Fig. 4C uncropped

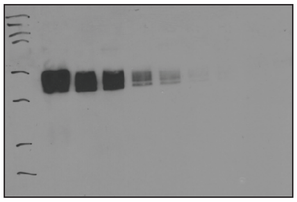

Figure S6. Fig. 4C, and the original uncropped images.

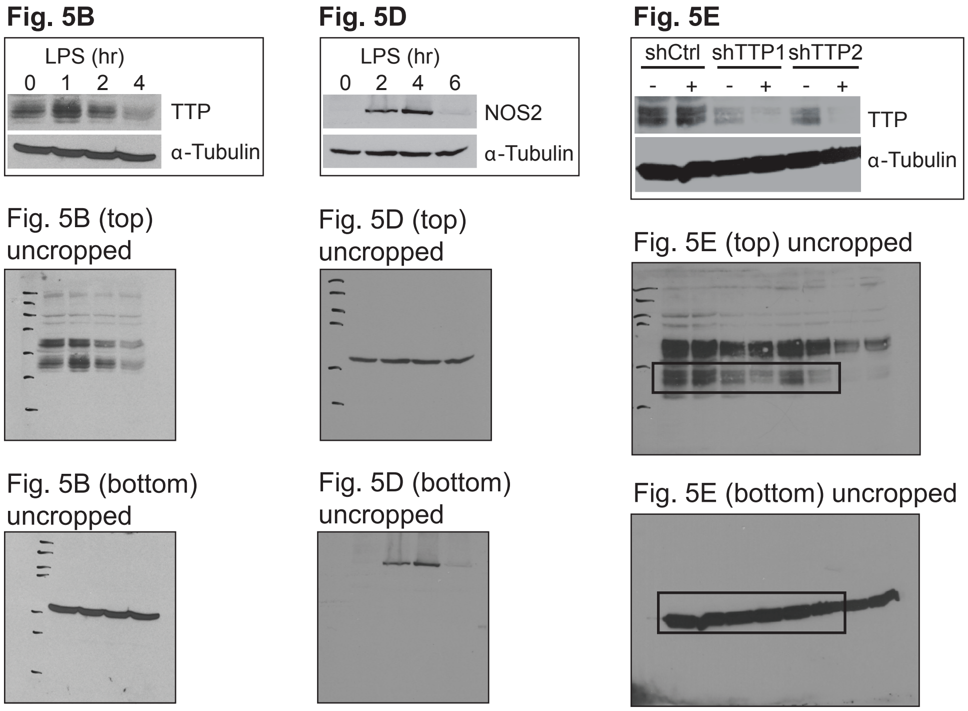

Figure S7. Fig. 5B, 5D, and 5E, and the original uncropped images. The lanes presented in Fig. 5E are boxed in the uncropped images.

Table S1: Differentially expressed genes in TTP knockout versus control colons

gene_id	gene	locus	log2FC	test_stat	p_value	q_value
ENSMUSG00000074448	Gm5615	9:36532396-36541963	4.1011	5.4147	5.00E-05	3.82E-03
ENSMUSG00000022061	Nkx3-1	14:69190637-69194662	4.0931	4.5731	5.00E-05	3.82E-03
ENSMUSG00000078776	9530053A07Rik	7:28129465-28164811	4.0187	5.4052	5.00E-05	3.82E-03
ENSMUSG00000041669	Prima1	12:103196907-103242150	3.8112	1.4420	9.50E-04	3.10E-02
ENSMUSG00000094356	Igkv8-28	6:70143592-70143897	3.6389	4.5519	5.00E-05	3.82E-03
ENSMUSG00000020826	Nos2	11:78920786-78960254	3.4427	5.6234	5.00E-05	3.82E-03
ENSMUSG00000094787	Ighv1-54	12:115193674-115194134	3.4109	3.5960	5.00E-05	3.82E-03
ENSMUSG00000098470	C1rb	6:124570293-124581171	3.2582	3.3198	4.00E-04	1.78E-02
ENSMUSG00000015619	Gata3	2:9857077-9890034	3.0562	1.4201	1.50E-04	8.64E-03
ENSMUSG00000052430	Bmpr1b	3:141837135-142169425	3.0125	1.8879	5.00E-05	3.82E-03
ENSMUSG00000023263	9530002B09Rik	4:122689274-122705129	2.9520	4.8197	5.00E-05	3.82E-03
ENSMUSG00000035896	Rnase1	14:51145001-51146767	2.8546	3.6048	5.00E-05	3.82E-03
ENSMUSG00000094951	Ighv5-6	12:113625505-113625956	2.7917	4.0774	5.00E-05	3.82E-03
ENSMUSG00000027820	Mme	3:63241536-63386030	2.5785	1.7931	5.00E-05	3.82E-03
ENSMUSG00000031594	Fgl1	8:41191433-41215156	2.4963	2.6488	5.50E-04	2.14E-02
ENSMUSG00000035934	Pknox2	9:36890981-37147322	2.4588	1.1040	1.00E-04	6.43E-03
ENSMUSG00000060093	Hist1h4a	13:23760691-23761230	2.2115	2.7019	3.50E-04	1.65E-02
ENSMUSG00000095429	Ighv5-12	12:113702123-113702578	2.1443	2.1918	1.55E-03	4.25E-02
ENSMUSG00000031273	Col4a6	X:141165402-141474076	2.1009	2.2810	5.00E-05	3.82E-03
ENSMUSG00000015053	Gata2	6:88193890-88207030	2.0984	2.8513	5.00E-05	3.82E-03
ENSMUSG00000024401	Tnf	17:35199380-35202007	2.0771	2.9420	5.00E-05	3.82E-03
ENSMUSG00000095981	Ighv10-1	12:114479004-114479451	1.9897	3.4364	5.00E-05	3.82E-03
ENSMUSG00000095197	Ighv1-59	12:115335081-115335514	1.9726	2.4082	4.50E-04	1.90E-02
ENSMUSG00000042671	Rgs8	1:153653024-153700323	1.9637	0.8151	1.15E-03	3.52E-02
ENSMUSG00000093908	Gm5784	12:19387808-19390286	1.9415	2.7833	1.00E-04	6.43E-03
ENSMUSG00000019817	Plagl1	10:13060503-13131694	1.9148	0.3888	5.00E-05	3.82E-03
ENSMUSG00000095612	Ighv5-4	12:113597445-113597977	1.8732	2.9842	5.00E-05	3.82E-03
ENSMUSG00000062727	Hist1h2bk	13:22035869-22036345	1.8339	3.0390	5.00E-05	3.82E-03
ENSMUSG00000103373	RP24-270A10.5	13:48542643-48545033	1.8116	2.6630	1.00E-04	6.43E-03
ENSMUSG00000096807	Hist1h2bm	13:21722097-21722478	1.8062	2.5162	1.50E-04	8.64E-03
ENSMUSG00000041779	Tram2	1:20996298-21079229	1.8016	1.9728	7.00E-04	2.51E-02
ENSMUSG00000076731	Ighv8-12	12:115647960-115648361	1.7948	2.9596	5.00E-05	3.82E-03
ENSMUSG00000026773	Pfkfb3	2:11471432-11554077	1.7947	2.4293	5.00E-05	3.82E-03
ENSMUSG00000080316	Spaca6	17:17830718-17843009	1.7841	2.1698	1.05E-03	3.31E-02
ENSMUSG00000072621	Slfn10-ps	11:83028129-83040042	1.7750	1.9870	5.50E-04	2.14E-02
ENSMUSG00000068606	Gm4841	18:60268300-60273267	1.7684	2.3776	1.00E-04	6.43E-03
ENSMUSG00000095589	Ighv1-55	12:115208094-115208591	1.7558	2.2902	5.00E-04	2.03E-02
ENSMUSG00000017204	Gsdma	11:98664350-98677708	1.7384	2.8439	5.00E-05	3.82E-03
ENSMUSG00000076583	Igkv8-24	6:70216857-70217421	1.7199	2.4516	1.00E-04	6.43E-03
ENSMUSG00000076549	Igkv4-68	6:69304833-69305363	1.7194	2.4473	5.00E-05	3.82E-03
ENSMUSG00000069267	Hist1h3b	13:23752266-23752886	1.6942	2.8188	5.00E-05	3.82E-03
ENSMUSG00000060678	Hist1h4c	13:23698059-23698454	1.6725	3.5048	5.00E-05	3.82E-03
ENSMUSG00000088529	Gm26083	11:117076789-117078955	1.6688	2.5805	5.00E-05	3.82E-03
ENSMUSG00000057280	Musk	4:58285959-58374303	1.6593	1.8200	3.50E-04	1.65E-02
ENSMUSG00000069268	Hist1h2bf	13:23573735-23574196	1.6575	3.3204	5.00E-05	3.82E-03
ENSMUSG00000054072	Iigp1	18:60376028-60392627	1.6574	2.4270	1.50E-04	8.64E-03
ENSMUSG00000069301	Hist1h2ag	13:22042459-22042944	1.6514	2.5025	1.50E-04	8.64E-03
ENSMUSG00000037053	Azgp1	5:137981520-137990233	1.6372	2.1412	6.00E-04	2.27E-02
ENSMUSG00000069266	Hist1h4b	13:23756202-23757620	1.6325	2.3706	1.45E-03	4.05E-02
ENSMUSG00000028017	Egf	3:129677574-129755322	1.6065	2.7989	5.00E-05	3.82E-03
ENSMUSG00000092837	Rpph1	14:50807448-50807767	1.5946	2.9299	5.00E-05	3.82E-03
ENSMUSG00000050936	Hist2h2bb	3:96267074-96279001	1.5928	3.0179	5.00E-05	3.82E-03

ENSMUSG00000028613	Lrp8	4:107802260-107876840	1.5775	1.6064	1.45E-03	4.05E-02
ENSMUSG00000037552	Plekhg2	7:28359603-28372599	1.5648	2.0610	2.00E-04	1.09E-02
ENSMUSG00000078763	Slfn1	11:83116848-83122670	1.5617	1.8894	1.40E-03	3.97E-02
ENSMUSG00000049539	Hist1h1a	13:23763716-23764358	1.5566	2.9485	5.00E-05	3.82E-03
ENSMUSG00000097324	Mir143hg	18:61649624-61665537	1.5467	2.2142	4.00E-04	1.78E-02
ENSMUSG00000102365	RP23-114L12.1	1:138960440-138962903	1.5420	2.1374	9.50E-04	3.10E-02
ENSMUSG00000094694	Ighv1-9	12:114583568-114584002	1.5376	3.0065	5.00E-05	3.82E-03
ENSMUSG00000102746	RP23-255H8.1	3:67684358-67684752	1.5279	2.5921	1.00E-04	6.43E-03
ENSMUSG00000056018	1700008F21Rik	8:129067133-129183732	1.5277	1.8924	4.00E-04	1.78E-02
ENSMUSG00000054404	Slfn5	11:82910549-82962941	1.5155	2.3330	5.00E-05	3.82E-03
ENSMUSG00000060981	Hist1h4h	13:23531049-23531522	1.5039	2.8476	5.00E-05	3.82E-03
ENSMUSG00000067455	Hist1h4j	13:21735063-21735837	1.5032	2.3192	4.50E-04	1.90E-02
ENSMUSG00000046804	Phgr1	2:118772768-118778165	-1.5051	-3.6050	5.00E-05	3.82E-03
ENSMUSG00000006360	Crip1	12:113152011-113153877	-1.5193	-3.4816	5.00E-05	3.82E-03
ENSMUSG00000069792	Wfdc17	11:83703990-83706268	-1.5234	-2.2631	4.50E-04	1.90E-02
ENSMUSG00000055193	Klk15	7:43933770-43939590	-1.5593	-2.4467	1.50E-04	8.64E-03
ENSMUSG00000029373	Pf4	5:90772434-90773381	-1.5674	-2.4937	1.00E-04	6.43E-03
ENSMUSG00000028195	Cyr61	3:145646970-145649985	-1.5790	-3.1183	5.00E-05	3.82E-03
ENSMUSG00000002992	Apoc2	7:19671578-19681423	-1.5794	-2.2943	1.00E-04	6.43E-03
ENSMUSG00000023034	Nr4a1	15:101266845-101274792	-1.5819	-3.1912	5.00E-05	3.82E-03
ENSMUSG00000074218	Cox7a1	7:30184170-30186028	-1.6068	-3.3787	5.00E-05	3.82E-03
ENSMUSG00000062742	Gm5239	18:35536582-35536951	-1.6406	-2.3663	2.00E-04	1.09E-02
ENSMUSG00000034892	Rps29	12:69157721-69159186	-1.6492	-3.9137	5.00E-05	3.82E-03
ENSMUSG00000066362	Rps13-ps1	8:87047688-87048226	-1.6747	-3.1326	5.00E-05	3.82E-03
ENSMUSG00000095007	Igkv12-41	6:69858419-69858884	-1.6954	-2.2656	9.50E-04	3.10E-02
ENSMUSG00000017316	Ppy	11:102099929-102101319	-1.7066	-2.6371	1.50E-04	8.64E-03
ENSMUSG00000016427	Ndufa1	X:37187587-37191163	-1.7289	-3.9679	5.00E-05	3.82E-03
ENSMUSG00000062124	Defb45	2:152593190-152599399	-1.7322	-2.3660	5.00E-04	2.03E-02
ENSMUSG00000037185	Krt80	15:101349570-101370125	-1.7360	-2.1526	1.55E-03	4.25E-02
ENSMUSG00000042293	Gm5617	9:48495344-48495964	-1.7557	-2.4326	4.50E-04	1.90E-02
ENSMUSG00000031722	Hp	8:109575129-109579172	-1.7651	-2.3056	1.05E-03	3.31E-02
ENSMUSG00000062515	Fabp4	3:10204087-10208576	-1.8664	-3.6039	5.00E-05	3.82E-03
ENSMUSG00000060615	Ang4	14:51763877-51773590	-1.8727	-3.4700	5.00E-05	3.82E-03
ENSMUSG00000061780	Cfd	10:79890852-79892656	-1.8832	-3.3353	5.00E-05	3.82E-03
ENSMUSG00000103421	Golt1a	1:133309799-133329901	-1.9251	-2.0221	6.50E-04	2.37E-02
ENSMUSG00000020405	Fabp6	11:43596048-43601540	-1.9411	-3.5667	5.00E-05	3.82E-03
ENSMUSG00000041845	Rhod	19:4425458-4477447	-1.9939	-3.3901	5.00E-05	3.82E-03
ENSMUSG00000024912	Fosl1	19:5447702-5455945	-2.0189	-2.4784	6.50E-04	2.37E-02
ENSMUSG00000035042	Ccl5	11:83525777-83530518	-2.1077	-2.6354	1.00E-04	6.43E-03
ENSMUSG00000096594	Igkv8-19	6:70340875-70341180	-2.1890	-2.3935	5.50E-04	2.14E-02
ENSMUSG00000076576	Igkv6-32	6:70074023-70074584	-2.2453	-2.5685	2.50E-04	1.27E-02
ENSMUSG00000022650	Retnlb	16:48816855-48818891	-2.2793	-4.3098	5.00E-05	3.82E-03
ENSMUSG00000098892	Comt	CHR_MG3833_PATCH:18348181-18479958	-2.3128	-3.8233	5.00E-05	3.82E-03
ENSMUSG00000076577	Igkv8-30	6:70117060-70117617	-2.3723	-3.1964	5.00E-05	3.82E-03
ENSMUSG00000000326	Comt	16:18348181-18479073	-2.4547	-3.9553	5.00E-05	3.82E-03
ENSMUSG00000075279	Mrpl23-ps1	2:74591329-74591909	-2.5643	-3.5490	5.00E-05	3.82E-03
ENSMUSG00000100595	Gm19087	1:151107914-151108860	-2.6890	-4.4486	5.00E-05	3.82E-03
ENSMUSG00000076680	Ighv6-6	12:114434787-114435244	-2.7026	-3.3402	5.00E-05	3.82E-03
ENSMUSG00000095351	Igkv3-2	6:70698467-70699067	-3.1425	-4.4904	5.00E-05	3.82E-03
ENSMUSG00000044786	Zfp36	7:28376783-28379255	-4.7909	-8.7922	5.00E-05	3.82E-03

Table S2: Primer and shRNA sequences

Genotyping of Tail Biopsies	
Vil_1F	GCCTTCTCCTCTAGGCTCGT
Vil_2R	TATAGGGCAGAGCTGGAGGA
Vil_3R	AGGCAAATTTTGGTGTACGG
TTP_floxA_F	GAACCCTCTCTCGATCGGGGATAC
TTP_floxB_R	GGATGGAGTCCGAGTTTATGTTCCAA

Genotyping of Colon Tissue	
TTP_flox1_F	CTGGCTGGAAATGAGAGAGG
TTP_flox2_R	GGATGGAGTCCGAGTTTATGTTCCAA
TTP_flox3_R	CACCCCTTACGCCAGAACTA

RT-qPCR	
mZfp36_exp1_F	GGCAGGTCCCTAGTTTGCAAATTCA
mZfp36_exp1_R	AACAGAAGACTTGGGGCCTGGAAG
mActb_exp1_F	ATGTCACGCACGATTTCCCTCTC
mActb_exp1_R	CTATGCTCTCCCTCACGCCATCCT
mZfp36l1_exp1_F	TTAAGCTCAGCCCCTTTCCCAGAA
mZfp36l1_exp1_R	TGGGCTATGAAATGGAGGGAGAGG
mZfp36l2_exp1_F	GCAGCTACCAACCACCATTCCATC
mZfp36l2_exp1_R	GCTGGCTCACACTGGTCTTGGAAC
mTnf_exp1_F	CTCAGAGCCCCCAGTCTGTA
mTnf_exp1_R	CAGGTCACTGTCCCAGCATC
mCsf2_exp1_F	CTCACCCATCACTGTCACCC
mCsf2_exp1_R	AAATTGCCCCGTAGACCCTG
mIl6_exp2_F	AGCCAGAGTCCTTCAGAGAGA
mIl6_exp2_R	TGGTCTTGGTCCTTAGCCAC
mCcl2_exp1_F	GCAGGTGTCCCAAAGAAGCT
mCcl2_exp1_R	TGCTTGAGGTGGTTGTGGAA
mCxcl1_exp1_F	ACACAGCACCATGATCCCAG
mCxcl1_exp1_R	AGCTTCAGGGTCAAGGCAAG
mMyc_exp1_F	AGGACTGTATGTGGAGCGGTTTCT
mMyc_exp1_R	AGGCTGGAGGTGGAGCAGACG
mVegfa_exp1_F	TTCGGGAACCAGACCTCTCA
mVegfa_exp1_R	GACCCAAAGTGCTCCTCGAA
mNos2_exp1_F	CTCCCTTTCCTCCCTTCTTCTCCA
mNos2_exp1_R	CCCAAAGTGCTTCAGTCAGGAGGT
rZfp36_exp1_F	GTTCAGTGTCCGGTGGTTGT
rZfp36_exp1_R	CGTGGGGGAAAAGGGATTTG
rActb_exp1_F	GCTCTATCCTGGCCTCACTGTCCA
rActb_exp1_R	CCGGACTCATCGTACTCCTGCTTG
rTnf_exp1_F	AACACACGAGACGCTGAAGT
rTnf_exp1_R	TCCAGTGAGTTCCGAAAGCC
rNos2_exp1_F	TGGCCCTAAATAAAATGACAGTGAGG
rNos2_exp1_R	GAGGGGAGATGATGTGAGGGGTTT
Luc_exp1_F	CTGCAACACCCCAACATCTTCGAC
Luc_exp1_R	TGGCCACATAGTCCACGATCTCCT
Ren_exp1_F	GAAGAGGGCGAGAAAATGGTGCTT
Ren_exp1_R	AGCGAACTCCTCAGGCTCCAGTTT

Generation of Biotinylated Transcripts	
hTNF_bio_F1	CCAAGCTTCTAATACGACTCACTATAGGGAGAAGGACGAACATCCAACCTTC
hTNF_bio_R	TACGTCGACTTTCTCGCCACTGAATAGTAGG
mPlagl1_bio_F	CCAAGCTTCTAATACGACTCACTATAGGGAGATGGACAGTTTAGTTCCTCTTCTTG
mPlagl1_bio_R	GGTTTTACACTTTCAAAATCGATT
mIigp1_bio_F	CCAAGCTTCTAATACGACTCACTATAGGGAGAAATCGTCAACTGTGTATTTGGAGA
mIigp1_bio_R	TCAAGGATTCTGACTAAATAGGATTT
mEgf_bio_F	CCAAGCTTCTAATACGACTCACTATAGGGAGAACCCCTGAATAAATGATGTGATCT
mEgf_bio_R	CTGATGCTTTAGGGTGATTTTATT
mLrp8_bio_F	CCAAGCTTCTAATACGACTCACTATAGGGAGAACTGCTGAGTGACTAACATGATGC
mLrp8_bio_R	CGTGCCTCTTCTCTACAGATTTTT
mGm5615_bio_F	CCAAGCTTCTAATACGACTCACTATAGGGAGATGTGTCCTACTAGCTCCATCTCTG
mGm5615_bio_R	ATTTAGGACGCTGAAGAGAAAAGA
mNkx3-1_bio_F	CCAAGCTTCTAATACGACTCACTATAGGGAGAAATACTCAGCATTAAATCCCAAGC
mNkx3-1_bio_R	CTATCAACTTCCTTTGGCAGTTTT
mGata3_bio_F	CCAAGCTTCTAATACGACTCACTATAGGGAGAAGTTTTGTTTCCCTTCAGTTGTTT
mGata3_bio_R	AAAGTGACGAAAAAGACAGGTACC
mBmpr1b_bio_F	CCAAGCTTCTAATACGACTCACTATAGGGAGAGTAAAGAAAAGCATCCCTCTGTGT
mBmpr1b_bio_R	TCATGGATTACTGGAAAGTCAACA
mMme_bio_F	CCAAGCTTCTAATACGACTCACTATAGGGAGAATTTTGTCTTTCAGTCATGCATTA
mMme_bio_R	CAACAGCTCTCAAGTGTACAACAA
mGapdh_bio_F1	CCAAGCTTCTAATACGACTCACTATAGGGAGAGAAACCCTGGACCACCC
mGapdh_bio_R	GATGTCGACGGGTGCAGCGAACTTTATTG
mNos2_bio_F2	CCAAGCTTCTAATACGACTCACTATAGGGAGAACTCAACCTCCTGACTGAAGC
mNos2_bio_R	GACGTCGACCTATAAAACTTGACCAAACACTCAGG

Generation of Luciferase Constructs	
hTNF_3luc_F	CTAGAGCTCAGGACGAACATCCAACCTTC
hTNF_3luc_R	TACGTCGACTTTCTCGCCACTGAATAGTAGG
mNos2_3luc_F1	CTAGAGCTCCAGCCCAGAGTTCCAGCT
mNos2_3luc_F2	CTGGAGCTCACTCAACCTCCTGACTGAAGC
mNos2_3luc_R2	TATGTCGACGCTTCAGTCAGGAGGTTGAGT
mNos2_3luc_R1	GACGTCGACCTATAAAACTTGACCAAACACTCAGG
mNos2_QC1_F	TCATCTTTCAGAAAACAGATACTTTTGTCTAC
mNos2_QC1_R	AGTAGAAAGATCTGTACATAGTGCAGC
mNos2_QC2_F	GATGAAAAATCTTTCTATAAAATACATTTTATTTTAATCAC
mNos2_QC2_R	AAGGAATTATACAGGAAAGG
mGapdh_3luc_F	CAAGAGCTCGAAACCCTGGACCACCC
mGapdh_3luc_R	GATGTCGACGGGTGCAGCGAACTTTATTG

Inducible Lentiviral shRNAs (Catalog #; target sequence)	
shCtrl	VSC11651; TGGTTTACATGTTGTGTGA
shTTP1	V3SR11254-240134005; TAATCATCAGGGTCGGATC
shTTP2	V3SR11254-242824792; TATGTTCCAAAGTCCTCCG
